# Characterization of Linkage Disequilibrium and Population Structure in a Mungbean Diversity Panel

**DOI:** 10.3389/fpls.2017.02102

**Published:** 2018-01-12

**Authors:** Thomas J. Noble, Yongfu Tao, Emma S. Mace, Brett Williams, David R. Jordan, Colin A. Douglas, Sagadevan G. Mundree

**Affiliations:** ^1^Centre for Tropical Crops and Biocommodities, Queensland University of Technology, Brisbane, QLD, Australia; ^2^Queensland Alliance for Agriculture and Food Innovation, University of Queensland, Warwick, QLD, Australia; ^3^Department of Agriculture and Fisheries, Hermitage Research Facility, Warwick, QLD, Australia

**Keywords:** mungbean, *Vigna radiata*, genetic diversity, linkage disequilibrium, population structure, genome-wide association mapping, SNP

## Abstract

Mungbean [*Vigna radiata* (L.) R. Wilczek var. *radiata*] is an important grain legume globally, providing a high-quality plant protein source largely produced and consumed in South and East Asia. This study aimed to characterize a mungbean diversity panel consisting of 466 cultivated accessions and demonstrate its utility by conducting a pilot genome-wide association study of seed coat color. In addition 16 wild accessions were genotyped for comparison and in total over 22,000 polymorphic genome-wide SNPs were identified and used to analyze the genetic diversity, population structure, linkage disequilibrium (LD) of mungbean. Polymorphism was lower in the cultivated accessions in comparison to the wild accessions, with average polymorphism information content values 0.174, versus 0.305 in wild mungbean. LD decayed in ∼100 kb in cultivated lines, a distance higher than the linkage decay of ∼60 kb estimated in wild mungbean. Four distinct subgroups were identified within the cultivated lines, which broadly corresponded to geographic origin and seed characteristics. In a pilot genome-wide association mapping study of seed coat color, five genomic regions associated were identified, two of which were close to seed coat color genes in other species. This mungbean diversity panel constitutes a valuable resource for genetic dissection of important agronomical traits to accelerate mungbean breeding.

## Introduction

Mungbean [*Vigna radiata* (L.) R. Wilczek var. *radiata*] is a grain legume originating from South Asia. It belongs to subgenus *Ceratotropis* of the Fabaceae family which comprises 23 species including the closely related wild mungbean [*Vigna radiata* var. *sublobata* (Roxb) Verdcourt]. As a summer legume species with a short growth duration (55–70 days from sowing to maturity) and the ability to fix atmospheric nitrogen, mungbean delivers economic, farming systems, and environmental benefits. The crop is vital to smallholder farmers in Asia with an annual production of 3.5–4.0 million tons where it represents an excellent cheap source of carbohydrates, high quality protein (dry seeds 27% protein), folate and iron (Day, 2013). Over the last three decades, the global consumption of mungbean has increased by 60% with a corresponding growth in production area up to 6 million hectares, concentrated mainly in South, East, and Southeast Asia (Kim et al., 2015). As an orphan crop of subsistence agriculture with limited genetic information available, mungbean improvement has relied on traditional plant breeding methodologies for most of its cultivated history (Fernandez et al., 1988; Humphry et al., 2002). In fact investment to-date in the development of new mungbean varieties has been low, resulting in a narrow genetic base of the crop leaving the crop vulnerable to many abiotic and biotic stresses. Additionally, progress in developing genomics tools to support molecular breeding activities for developing improved mungbean varieties has been very limited. However, the recent release of a reference genome for mungbean (Kang et al., 2014) provides new opportunities for mungbean genomic research (Kim et al., 2015).

Mungbean is a self-pollinated diploid species with a chromosome number of 2n = 22 with an estimated genome size of 543 mega bases (Mb) (Parida et al., 1990; Kang et al., 2014). The relatively small genome size makes it an attractive and valuable model for advancing the understanding of diversity and evolution of legume genomes. The domestication of mungbean is considered to have taken place approximately 3500 years ago (Kim et al., 2015) from a small number of elite founders. The domestication process has resulted in significant genetic bottlenecks in the cultivated mungbean genome (Lakhanpaul et al., 2000). Previous genetic diversity studies of cultivated and wild mungbean germplasm, using both morphological and molecular markers, have highlighted low levels of genetic diversity in cultivated mungbean compared to the broader diversity found in wild mungbean (Santalla et al., 1998; Saravanakumar et al., 2004; Sangiri et al., 2007).

Wild mungbean relatives are of critical importance to broaden the genetic diversity of cultivated mungbean given the important biotic and abiotic stress resistance genes they may contain, which have not yet been incorporated into cultivated material by breeding programs.

In fact the use of wild relatives to increase productivity has been successfully demonstrated in many other crops including sorghum (Jordan et al., 2011) and barley (Ellis et al., 2000). Previous studies of genetic variation in wild mungbean germplasm have reported valuable genes affecting important agronomical traits such as phenology (Rebetzke and Lawn, 2006a); growth, biomass, and seed yield (Rebetzke and Lawn, 2006b); root and shoot attributes (Rebetzke and Lawn, 2006c). These genes could be introgressed into cultivated mungbeans to increase the resilience of cultivated material and broaden the overall genetic base (Pandiyan et al., 2008). Introgression of traits from closely related wild relatives into cultivated mungbean has been reported to produce fertile progeny with favorable morphological characteristics (Lambrides et al., 1999). By contrast, *Vigna mungo* [*V. mungo* (L.) Hepper], or black gram, produces sterile progeny when hybridized to mungbean (Lambrides et al., 1999).

Dissecting the genetic basis of important agronomic traits, such as seed coat color, grain size, flowering time and disease resistance, are critical to manipulating and introgressing these traits precisely to achieve breeding goals. Traditionally, linkage mapping is the primary tool to identify genetic loci underlying traits of interest. A limited number of genetic linkage maps have been developed in mungbean (Lambrides et al., 2000; Humphry et al., 2002; Isemura et al., 2012), however, despite great efforts a comprehensive and saturated genetic linkage map of all 11 chromosomes has not been generated (Kim et al., 2015). Instead, high density maps developed from whole genome sequences (Kang et al., 2014) enable further advancement in alternative approaches to trait dissection, such as association mapping, also known as linkage disequilibrium (LD) mapping (Gupta et al., 2005; Abdurakhmonov and Abdukarimov, 2008). LD mapping takes advantage of historical recombination events in a diverse set of lines to identify the genetic basis of traits at a higher resolution than traditional genetic linkage mapping. The resolution of association mapping relies upon the extent of LD. The degree of LD has yet to be accurately determined in mungbean. In cultivated material of the closely related species soybean, high levels of LD have been reported (∼150 kb), potenitally caused by inbreeding, adaptation and human selection (Lam et al., 2010). By contrast LD in wild soybean species was considerably lower (∼75 kb), indicating wild germplasm could play a major role in influencing the dynamics of genome change.

This study aims to assess the genetic diversity, population structure, LD and mapping capabilities of a large and diverse mungbean germplasm panel using a high-throughput SNP genotyping platform. This study provides a unique genomic resource for genetic dissection of important agronomical traits to accelerate mungbean breeding.

## Materials and Methods

### Plant Materials

The mungbean diversity panel consists of 466 accessions representing the cultivated mungbean [*Vigna radiata* (L.) R. Wilczek var. *radiata*] germplasm held in Australia by the National Mungbean Improvement Program (Queensland, DAF). The material originated from various sources worldwide and represents the widest range of phenotypic traits observed and characterized by the mungbean breeding team over the past 50 years. These included seed coat color, seed size and weight, days to flower, days to maturity, plant habit, plant height, and reaction to key foliar diseases. Sixteen wild accessions [*Vigna radiata* var. *sublobata* (Roxb Verdcourt)] originating from Australia were also included in the study as a comparison to the diversty panel of cultivated mungbeans.

### Genotyping

Total genomic DNA was extracted from bulked young leaves of a single plant per accession as described by DArT P/L (DArT^1^). The samples were genotyped following an integrated DArT and genotyping-by-sequencing (GBS) methodology involving complexity reduction of the genomic DNA to remove repetitive sequences using methylation sensitive restrictive enzymes prior to sequencing on next generation sequencing platforms (DArT^2^). The sequence data generated were then aligned to the mungbean reference genome sequence, Vradi_ver6 (Kang et al., 2014), to identify single nucleotide polymorphisms (SNPs) markers.

### Phenotypic Data Collection

All 482 mungbean accessions were planted at Hermitage Research Facility, Warwick, QLD, Australia (28°12′ S, 152°5′ E), over the summer of 2015. The field trial design was unreplicated single field plots for each accession, 4.5 m^2^ in size containing an average of 130 plants. Seed coat color was qualitatively recorded based on five categories (green, black, brown, yellow, and speckled).

### Analysis of Genetic Diversity

The polymorphism information content (PIC) of each DArTseq SNP marker was determined using the following formula: PIC = 1-Σ(Pi^2^)/n where Pi is the frequency of the i th allele and n is the total number of genotypes (Weir, 1990).

### Estimation of Linkage Disequilibrium

The pairwise LD between SNPs genome-wide across the wild and cultivated mungbean genotypes was calculated based on the allele frequency correlations (*r^2^*) using the TASSEL program (v5.1.0) (Bradbury et al., 2007; Tao et al., 2013). Only *r^2^* for SNPs with pairwise distance less than 500 kb of each chromosome were used to draw the average LD decay figure. The LD decay graph was drawn by fitting a smooth spline of averaged *r^2^* over physical distance in R v3.3.1. The LD decay was calculated when the squared correlations of allele frequencies *r^2^* decreased to half of its maximum value.

### Analysis of Population Structure

Population structure of the 466 cultivated accessions was analyzed using a Structure-like Population Genetic Analyses in R package LEA (Falush et al., 2007). The number of subpopulations is determined using a cross-entropy criterion. The cross-entropy criterion is based on the prediction of a fraction of masked genotypes (matrix completion), and on the cross-validation approach. Smaller values of the cross-entropy criterion usually mean better runs. We perform runs for 9 values of *K* (*K* = 2:10), and choose the value of *K* for which the cross-entropy curve exhibits a plateau (*K* = 4).

Results of structure analysis were used to identify accessions representing the four sub-populations. An individual accession with more than 90% identity from a single sub-population was classified as representative of that sub-population. Genetic differentiation (Fixation index, F_ST_) among the four sub-populations was calculated using R package PopGenome (Pfeifer et al., 2014).

Genetic relationships among cultivated accessions were also analyzed using principal coordinate analysis (PCA) performed in the software package DARwin v6.0 (Perrier and Jacquemoud-Collet, 2006).

### Genome-Wide Association Mapping of Seed Coat Color

Association mapping was conducted using mixed linear model (MLM) controlling for Q and kinship (K) as fixed and random effects respectively in TASSEL 5.1.0. Minor allele frequency (MAF) > 0.01 was used to filter SNPs prior to analysis. Q was extracted from results of previous population structure analysis, which detected four sub-populations. *K* was calculated using Scaled IBS method implemented in TASSEL 5.1.0. Bonferroni correction was applied to set thresholds for *P*-value significant (0.05/n); n represented the number of SNPs used in trait-marker association analysis. The Manhattan plot was generated using the R package qqman (Turner, 2014).

## Results

### Germplasm Diversity Analysis

**FIGURE 1:**
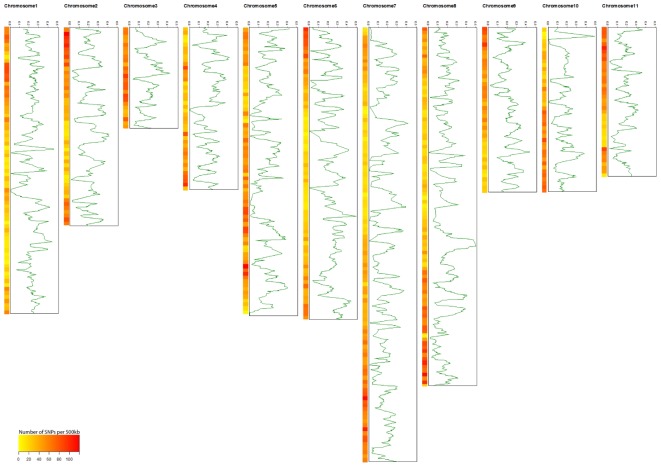
The distribution of polymorphism information content (PIC) genome-wide of 466 cultivated mungbean accessions. Heat maps on each chromosome represent SNP densities within a window of 500 kb.

A total of 22,230 SNP markers were identified in the cultivated and wild mungbean populations, of those 16,462 were physically mapped across the 11 mungbean chromosomes (Supplementary Table S1). An average of 1,497 SNPs were identified per chromosome (from 903 SNPs on chromosome 3 to 2,306 on chromosome 7) with an average marker density of 57.81 SNPs/Mb (**Table 1** and **Figure 1**). A total of 7,675 SNPs segregated within the cultivated population, with an average PIC value of 0.174. In contrast, 6,174 SNPs segregated within the wild population with an average PIC value of 0.305 (Supplementary Figure S1).

**Table 1:** Genomic distribution of 22,230 single nucleotide polymorphisms (SNPs) physically mapped on 11 cultivated and wild mungbean chromosomes/unanchored scaffolds.

Chromosome	Size of chromosome (Mb)	Total no. of SNPs	% of total SNPs	Avg. no. of markers per (Mb)	No. of cultivated SNPs	No. of wild SNPs
1	36.49	1643	7.39	45.03	835	267
2	25.34	1332	5.99	52.56	538	340
3	12.93	903	4.06	69.85	457	171
4	20.78	1044	4.70	50.23	512	219
5	37.09	1896	8.53	51.12	769	483
6	37.41	1666	7.49	44.53	851	278
7	55.45	2306	10.37	41.59	979	498
8	45.72	2204	9.91	48.21	1072	405
9	20.97	1208	5.43	57.60	638	181
10	20.99	1099	4.94	52.36	454	282
11	19.67	1161	5.22	59.03	570	239
Total	332.84	16462	74.05	NA	7675	3363
Average	30.26	1497	NA	52.01	698	306
Unanchored	NA	5768	25.95	NA	2423	1180

### Estimation of Linkage Disequilibrium

**FIGURE 2:**
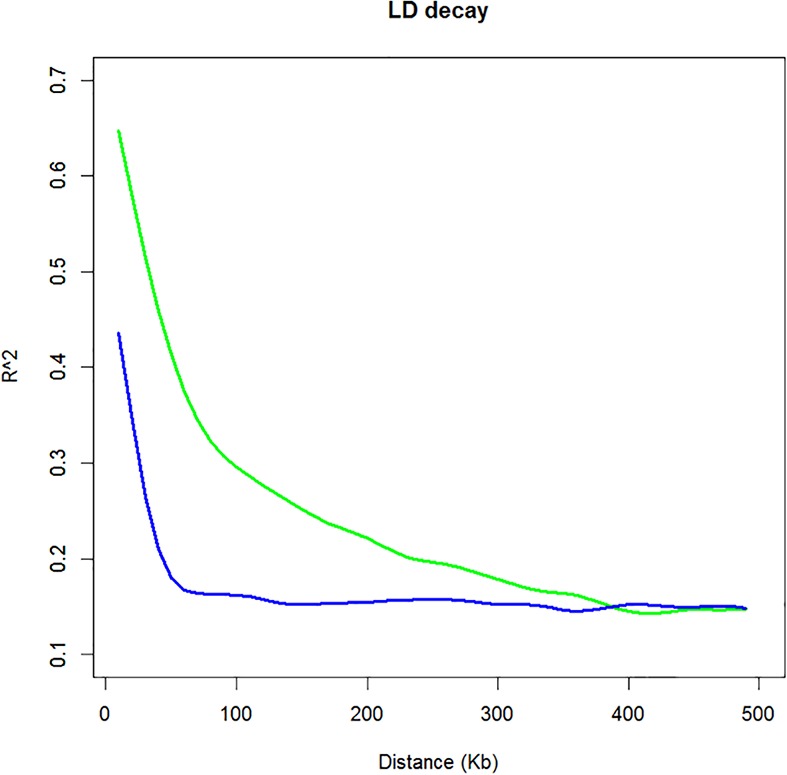
Linkage disequilibrium (LD) graph of cultivated and wild mungbean. LD is determined by squared correlations of allele frequencies (*r*^2^) against distance between polymorphic sites, color-coded as follows: cultivated accessions (green) and wild accessions (blue).

Linkage disequilibrium was estimated between all SNP markers over the 466 cultivated and 16 wild mungbean accessions. The squared correlations of allele frequencies *r^2^* of the cultivated mungbean population decreased to half of its maximum value at approximately 100 kb physical distance compared to the wild mungbean population which had largely decayed by 60 kb (**Figure 2**). Additionally, individual chromosomes of the cultivated population were estimated, no substantial differences were observed (Supplementary Figure S2).

### Population Structure of the Cultivated Population

**FIGURE 3:**
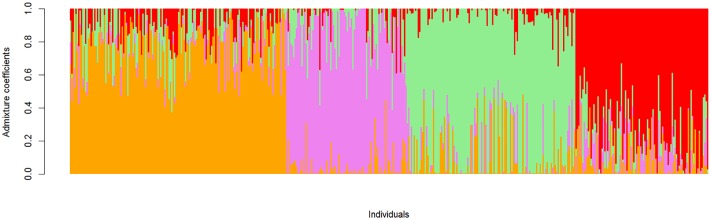
Population structure of 466 cultivated mungbean accessions at *K* = 4. Each vertical bar represents a single accession, the length of each bar represents the proportion contributed by each sub-population. Sub-population 1 (color-coded orange), sub-population 2 (color-coded pink), sub-population 3 (color-coded green), and sub-population 4 (color-coded red).

Structure-like population genetic analysis was used to analyze the structure of the cultivated population (**Figure 3**). A range of sub-populations (*K* = 2:10) were tested and a *K*-value of 4 was determined to best capture the structure of the cultivated population based on minimal cross-entropy (Supplementary Figure S3). Representative accessions from the four sub-populations defined as being > 90% relationship to a single sub-population were further characterized using phenotypic data (Supplementary Table S2).

Sub-population 1 was made up of 25 accessions, there was limited passport data as the majority of lines are Australian commercial lines (Crystal, Jade-AU) or Australian breeding lines (e.g., M11122). Those accessions with passport data were mostly from Southern Asia, Taiwan, Thailand, Philippines, and Vietnam with uniformly green seed coats.

Sub-population 2 was comprised of 40 accessions and had the widest range of passport data, with the accessions predominantly from Middle Eastern countries including Afghanistan, Uzbekistan, Tajikistan, Kyrgyzstan and Iran, with single accessions from China, India, Netherlands, and Turkey. This sub-population also had the widest range of seed coat colors, although largely green with a number of speckled, a single yellow accession and additionally this was the only sub-population to contain brown seed coats.

Sub-population 3 contained the largest number of accessions (59) mainly derived from Southern Asian countries, Cambodia, Philippines and Thailand with single accessions from China, Hong Kong, India, Indonesia, and Vietnam. This sub-population also contained the highest number of accessions from Iran (6). Of the 59 accessions, 19 displayed yellow seed coats while the remaining accessions were green in color with two speckled seed coats.

Sub-population 4 contained the lowest number of accessions (22), and the majority of the accessions did not have passport data; those that did were from India and Pakistan with a single line from Taiwan. Seed coat colors were primarily green with three accessions displaying speckled seed coats.

**FIGURE 4:**
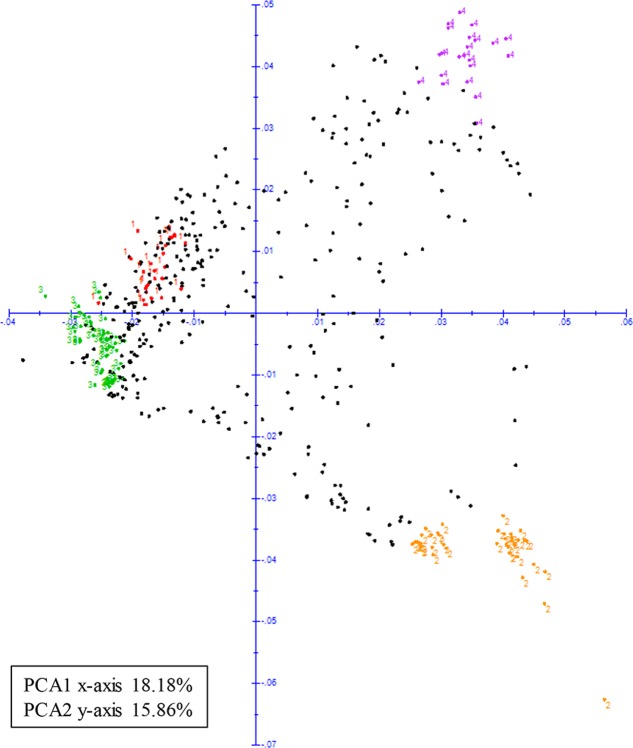
Principal coordinate analysis (PCA) of 466 cultivated mungbean genotypes. Color-coded according to membership (based on >90% identity) to sub-populations identified from structure analysis; sub-population 1 (color-coded red), sub-population 2 (color-coded yellow), sub-population 3 (color-coded green), and sub-population 4 (color-coded purple).

Principal coordinate analysis was also used to visualize the relationships amongst the cultivated accessions in the panel. When the four sub-populations were plotted they clustered toward the extremities of the plot based on their genetic differences (**Figure 4**). The first two principal coordinates accounted for approximately 34.04% of the genotypic variance with coordinates one (x-axis) and two (y-axis) explaining 18.18 and 15.86%, respectively.

### Genetic Diversity between Sub-populations

The genome-wide genetic differentiation between the four contrasting mungbean sub-populations identified in structure were calculated using fixation index (F_ST_) using PopGenome. Sub-populations 1 and 3 were the most closely related with an overall F_ST_ value of 0.42, while sub-populations 1 and 2 show the highest degree of differentiation, with an F_ST_ value of 0.57 (**Table 2**). Sub-population 1 had uniform green seed coats, in contrast to sub-population 2 which had a wide variation of seed coat colors. Sub-populations 1 and 3 however, shared similar characteristics with many accessions originating from Southern Asia and uniform seed color.

**Table 2:** Genome-wide genetic differentiation (F_ST_) between cultivated mungbean sub-populations.

	Pop2	Pop3	Pop4
Pop1	0.57	0.42	0.48
Pop2		0.56	0.52
Pop3	0.56		0.53

### Genome-Wide Association Study of Seed Coat Color QTL

TASSEL v5.1.0 was used to conduct a pilot genome-wide association mapping (GWAS) for seed coat color to demonstrate the effectiveness of the diversity panel for trait dissection. Seed coat color was chosen because it’s an oligogenic trait which does not change based on the environment the plant is grown and is economically important to the mungbean industry. Using the MLM model, nine SNPs were identified as significantly associated with seed color based on *P-value* < 9.66E-06 (Bonferroni correction). These SNPs were located in five distinct genomic regions distributed across chromosomes 3, 4, 5, and 7 (Supplementary Figure S5 and Supplementary Table S3). Five significant SNPs, including the most highly significant SNP overall, clustered within an interval of 22 kb on chromosome 4 between positions 17,668,384 and 17,690,573. Identified within this region VrMYB113 is the homolog of the Arabidopsis gene MYB113 involved in anthocyanin biosynthesis (Gonzalez et al., 2008). Vrsf3′h1 is the homolog of a previously identified gene (*sf3*′*h1*) controlling seed coat color through flavonoid 3′-hydroxylase in soybean (Toda et al., 2012) associated with a significant SNP on chromosome 5 at position 32413093. The most significant marker (6160927|F| 0–16:A > G-16:A > G) on chromosome 4 position 17679978 had a 97% association with lighter colored seed coats (green/yellow) on allele “1,” while allele “0” had a 91% association with darker pigmentation (black/speckled) (Supplementary Table S4).

## Discussion

The replacement of traditional and local crops with elite varieties harboring superior agronomic traits has led to increased yields but also the gradual erosion of genetic diversity of cultivated varieties. Globally, plant breeders are addressing the threat to cultivated crops with narrow genetic diversity by establishing large collections of genetically diverse germplasm. In this study, we investigated the application of genome-wide SNP markers to analyze the genetic diversity of a large, diverse mungbean germplasm panel. The markers generated from this germplasm panel provide a new resource to conduct high-resolution analysis of genetic diversity, population structure, LD and the capacity to identify genes controlling important agronomic traits. Additionally, this is the first high-resolution quantification of LD decay in mungbeans, defining the extent of LD within and between cultivated and wild mungbeans. The genetic diversity analysis, population structure and LD analysis provide the foundation that can be used to broaden the genetic base of mungbean breeding material. The genome-wide association study provides an example of how the data can be used to identify genomic regions responsible for phenotypic traits. SNP markers provide a level of resolution to breeding programs far beyond traditionally used methods which relied solely on passport data such as geographical origin and pedigrees (Brown, 1989) or genetic markers and quantitative phenotypic data to conduct cluster analysis of core collections (Bretting and Widrlechner, 1995). Developing effective, informed strategies for the utilization and management of germplasm collections is critical to mine the ready available diversity in mungbean (Fernandez et al., 1988). Application of this resource as a breeding tool has the potential to transform the improvement of this globally significant yet orphan crop through informed trait introgression from exotic germplasm and wild species using marker assisted breeding.

### Genetic Diversity of Cultivated Mungbean

The domestication syndrome, resulting from restricting the number of elite varieties used in breeding under selection for key traits such as larger grain size, has come at the expense of diminishing overall genetic diversity (Gepts, 2010). Low genetic diversity is impeding the rate of genetic gain that can be made in breeding programs through limiting the number of alleles available in breeding populations, severely restricting the ability of breeders to continue developing improved resistance and tolerance to biotic and abiotic stressors. Broadening the genetic base of mungbean and incorporating exotic alleles is essential to developing agile breeding programs that can respond to new threats and challenges faced by the crop (Nair et al., 2012). Over the last decade, limited research has been conducted to investigate the degree of genetic diversity within and between cultivated and wild mungbean. The first large-scale analysis of cultivated and wild mungbean germplasm using molecular markers was conducted by Sangiri et al. (2007) who analyzed the domestication of mungbean. Our study represents the first extensive mungbean germplasm diversity analysis using high-density SNP markers.

Highly informative PIC values as defined by Botstein et al. (1980) are values equal to or greater than 0.5 when considering multi-allelic markers whose values range from 0 to 1. PIC values for bi-allelic SNP markers range from 0 to 0.5, therefore we have regarded PIC values greater than or equal to 0.25 as highly informative. Within the cultivated population 34% of the SNPs had a PIC value greater than or equal to 0.25 compared to the wild population, which had 56%. The high PIC value derived from the wild population is consistent with our expectations that we would see a greater proportion of highly polymorphic markers in the wild population due to the selective breeding seen in the cultivated population.

The clear differentiation between cultivated and wild mungbean gene pools, as demonstrated in the PCA analysis (Supplementary Figure S4), was also reported by Sangiri et al. (2007), who noted close to double the gene diversity in the wild population in comparison to cultivated mungbeans and clear genetic differentiation among the cultivated accessions. Sangiri et al. (2007) observed that approximately 50% of the genetic variation present in the wild mungbean gene pool was found in the cultivated germplasm. In contrast, other cultivated cereals have been observed to contain only ∼30% of the genetic diversity present in wild species, indicating that mungbeans have experienced a weaker genetic bottle neck than that of the cereals, reflecting the difference in the domestication process of legumes compared with cereals (Buckler et al., 2001; Zhu et al., 2007).

### LD in Mungbean Is Comparable to Closely Related Species

The number and density of markers required for an association mapping analysis is determined by the distance over which LD decays. The LD patterns of mungbean reflect its long history of domestication (Fuller, 2007) and as a self-pollinated species the extent of genome-wide recombination is expected to be less than that observed in cross pollinated species (Flint-Garcia et al., 2003). The *r^2^* of cultivated mungbean decreased to half its maximum value at ∼100 kb compared to that of the wild species which decreased by ∼60 kb (**Figure 2**). Wild mungbean has retained a higher degree of allelic diversity providing an important source of material for increasing the genetic diversity of the cultivated gene pool.

Studies in closely related species report that LD largely decays by ∼150 kb in cultivated soybean; ∼75 kb in wild soybean (Lam et al., 2010) and in chickpea between 450 and 550 kb (Bajaj et al., 2015). In contrast, in *Medicago truncatula*, a leguminous plant used in genomic studies the LD decayed rapidly, dropping too approximately half of its initial value within ∼3 kb (Branca et al., 2011) similar to the model speices *Arabidopsis thaliana*: 3–4 kb (Kim et al., 2007). Cultivated and wild rice reported the highest variation between the highly cultivated varieties *O. japonica* at ∼200 kb and *O. indica* ∼65 kb in contrast to wild varieties *O. rufipogon* and *O. nivara* which exhibit rapid decay by ∼10 kb (Xu et al., 2011), similar to sorghum: improved inbreds ∼19.7 kb and land races ∼10.3 kb (Mace et al., 2013). These studies show the major influence domestication has had on changing genome dynamics and combined with PIC data, highlights the importance wild accessions and genetically diverse populations will have on providing access to new exotic alleles.

### High-Resolution Association Mapping Pilot Study

QTL (quantitative trait loci) are identified through the establishment of association between genomic marker data and traits of interest. Previous studies revealed the genetic factors controlling important agronomic traits in mungbean using biparental populations such as, major QTLs for hard-seededness and seed weight (Fatokun et al., 1992; Maughan et al., 1996; Humphry et al., 2005); powdery mildew resistance (Young et al., 1993; Chaitieng et al., 2002; Humphry et al., 2003; Kasettranan et al., 2010); yellow mosaic Indian virus resistance (Chen et al., 2012; Kitsanachandee et al., 2013); bruchid resistance (Young et al., 1992; Mei et al., 2009); *Cercospora* leaf spot resistance (Chankaew et al., 2011). Due to the limited number of recombination events (Balasubramanian et al., 2009), QTLs were commonly mapped to wide-ranging intervals extending over a number of centiMorgans (cM), usually megabases (Mb) long in physical distance, potentially containing 100s of candidate genes. Thus, the understanding of specific genes controlling agronomic traits is still restricted and further fine-mapping is often required to identify the underlying genes involved.

**FIGURE 5:**
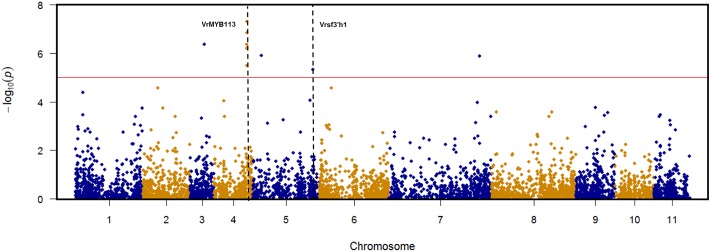
Genome-wide association mapping (GWAS) results for seed coat color in mungbean. Nine SNPs identified as significantly associated with seed color located in five distinct genomic regions distributed across chromosomes 3, 4, 5, and 7. VrMYB113 is the homolog of Arabidopsis gene MYB113 located on chromosome 4 between position 17,668,384 and 17,690,573. Vrsf3′h1 is the homolog of soybean gene sf3′h1 located on chromosome 5 position 32,413,093.

The structure of our population, which captured vast historical recombination accumulated during mungbean cultivation, combined with the high density of markers genome-wide overcome barriers seen in previous studies. The GWAS performed in this study (**Figure 5**) is an example of how the features of this data set can provide high resolution mapping opportunities. A Nested Association Mapping (NAM) population in mungbean, currently being developed by the Queensland mungbean improvement team, will provide a powerful complementary population to the diversity panel described here. The structure of the mungbean NAM population encompasses 26 of the most genetically and phenotypically diverse accessions, backcrossed to one elite line adapted to the local production (Supplementary Figure S5). environment. Robust precise phenotyping and genotying of the NAM population will push mungbean breeding years ahead of its time accelerating genome research and molecular breeding.

## Author Contributions

CD, DJ, and EM conceived and designed the experiments. TN collected the data. TN, EM, and YT analyzed data. TN, EM, and YT wrote the manuscript. SM, BW, DJ, and EM revised the manuscript. All authors read and approved the final manuscript.

## Conflict of Interest Statement

The authors declare that the research was conducted in the absence of any commercial or financial relationships that could be construed as a potential conflict of interest.

## Footnotes

1 http://www.diversityarrays.com

2 http://www.diversityarrays.com
