# Supplementary material for: Characterization of Linkage Disequilibrium and Population Structure in a Mungbean Diversity Panel
Source: Front Plant Sci. 2018 Jan 12;8:2102. doi: 10.3389/fpls.2017.02102 (PMC5770403; doi:10.3389/fpls.2017.02102)
Supplement: Supplementary file 5 [file Data_Sheet_1.DOCX]

Supplementary Material

Characterization of linkage disequilibrium and population structure in a mungbean diversity panel

Thomas Noble^1^*, Yongfu Tao^2^, Emma Mace^3^, Brett Williams^1^, David Jordan^2^, Alan Cruickshank^3^, Col Douglas^3^*, Sagadevan Mundree^1^*

^1^Centre for Tropical Crops and Bio Commodities (CTCB), Queensland University of Technology, Brisbane City, Queensland 4000, Australia

^2^ Queensland Alliance for Agriculture and Food Innovation (QAAFI), University of Queensland, Warwick, Queensland 4370, Australia

^3^Deptartment of Agriculture and Fisheries (DAF), Hermitage Research Facility, Warwick, Queensland 4370, Australia

*Correspondence

Thomas Noble

t2.noble@qut.edu.au

Sagadevan Mundree

[sagadevan.mundree@qut.edu.au](mailto:sagadevan.mundree@qut.edu.au)

Col Douglas

col.douglas@daf.qld.gov.au

**
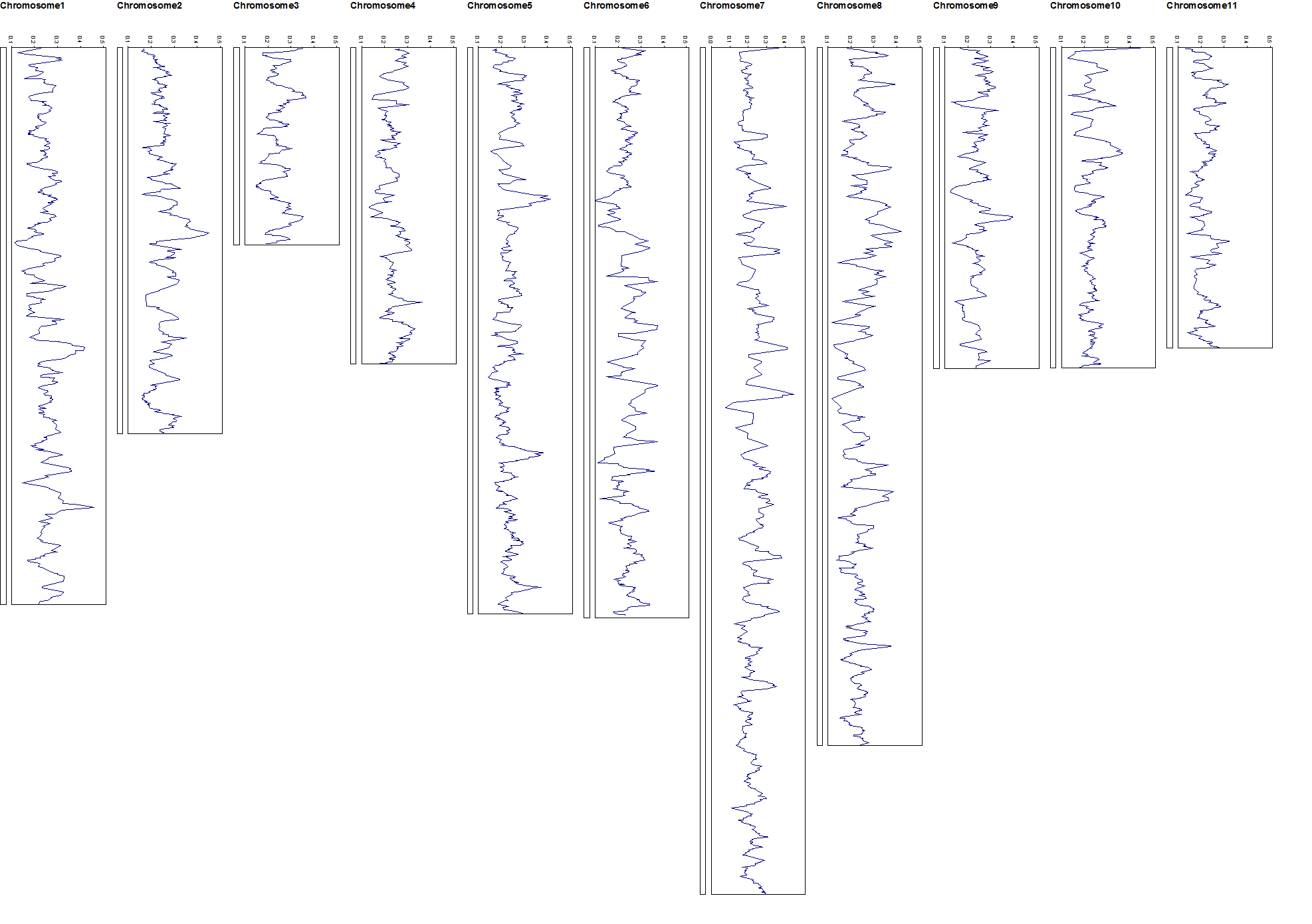

Figure S1.** The distribution of polymorphism information content (PIC) genome-wide of 16 wild mungbean accessions.


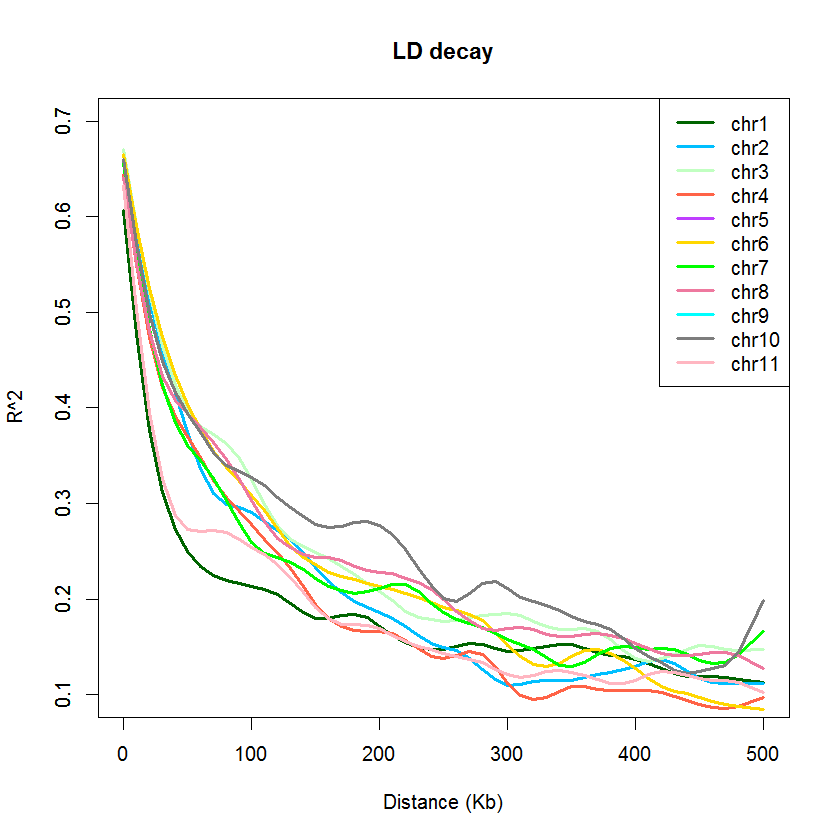


**Figure S2.** Linkage disequilibrium of individual chromosomes from cultivated mungbean. LD is determined by squared correlations of allele frequencies (*r*^2^) against distance between polymorphic sites, colour-coded as follows: cultivated accessions (green) and wild accessions (blue).


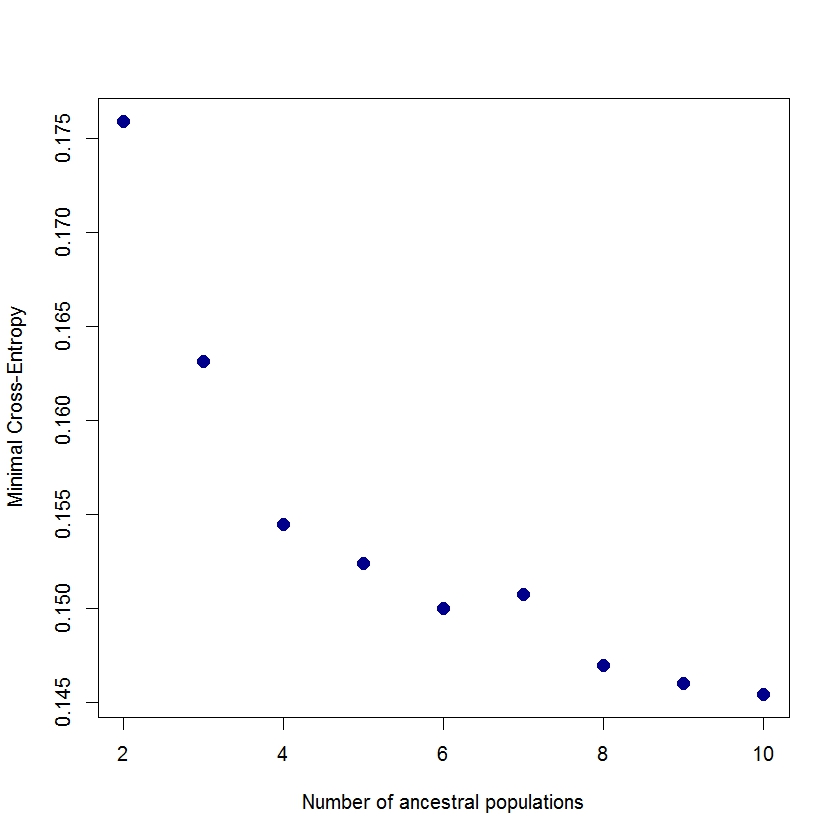


**Figure S3.** Cross-entropy plot for 466 mung bean diversity panel. X-axis indicates the number of ancestral populations, while Y-axis represents the minimal cross-entropy. A range of K=2:10 were tested, K=4 was chosen as the cross-entropy curve exhibits a plateau.


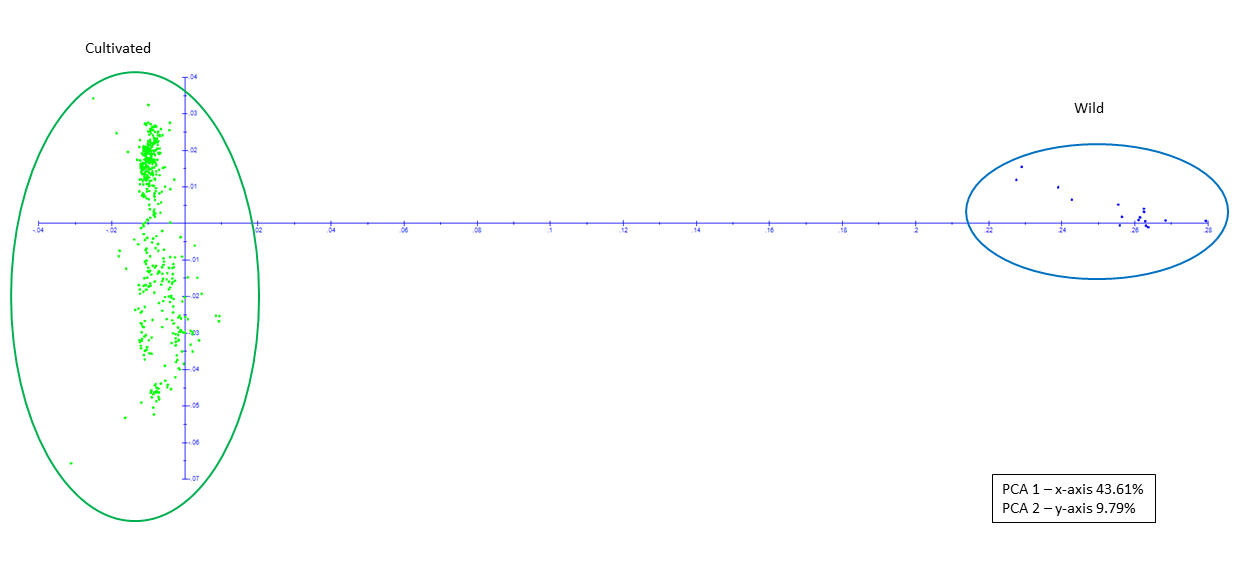


**Figure S4.** Principal coordinate analysis (PCA) of 466 cultivated mungbean accessions and 16 wild accessions, colour-coded as follows: cultivated accessions in green and wild accessions colour-coded red.

**
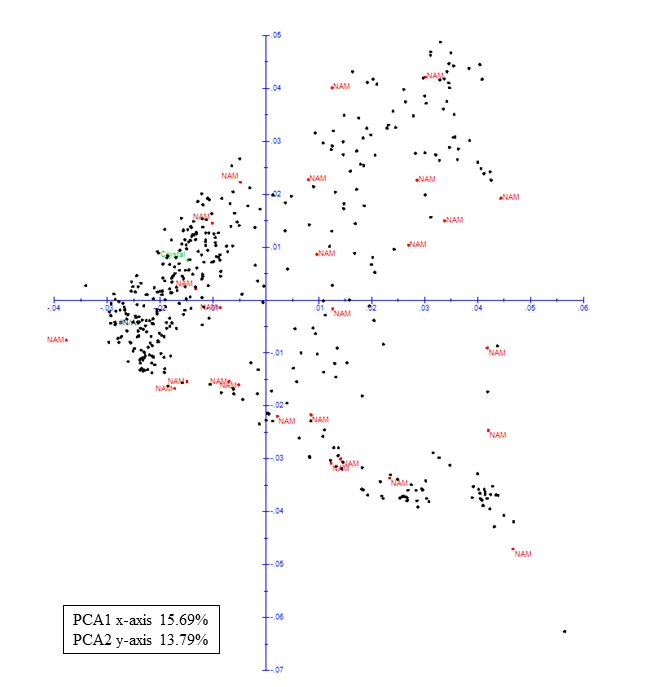
**

**Figure S5.** Principal coordinate analysis (PCA) of 466 cultivated mungbean genotypes. Twenty six accessions used as parental lines in the Nested Association Mapping (NAM) are highlighted in red and recurrent parent Crystal in is highlighted in green.
